# New Design for an Adjustable Cise Space Maintainer

**DOI:** 10.1155/2018/5434609

**Published:** 2018-04-30

**Authors:** Nurhat Ozkalayci, Mehmet Yetmez

**Affiliations:** ^1^Department of Orthodontics, Faculty of Dentistry, Bulent Ecevit University, 67100 Zonguldak, Turkey; ^2^Department of Mechanical Engineering, Faculty of Engineering, Bulent Ecevit University, 67100 Zonguldak, Turkey

## Abstract

**Objective:**

The aim of this study is to present a new adjustable Cise space maintainer for preventive orthodontic applications.

**Methods:**

Stainless steel based new design consists of six main components. In order to understand the major displacement and stress fields, structural analysis for the design is considered by using finite element method.

**Results:**

Similar to major displacement at *y*-axis, critical stresses **σ**_**x**_ and **τ**_**xy**_ possess a linear distribution with constant increasing. Additionally, strain energy density (SED) plays an important role to determine critical biting load capacity.

**Conclusion:**

Structural analysis shows that the space maintainer is stable and is used for maintaining and/or regaining the space which arouses early loss of molar tooth.

## 1. Introduction

Early loss of deciduous tooth or teeth causes space problem due to the crowding and/or unerupted tooth [1, 2]. Consequently, space maintenance is such a tool-based process that plays important role in preventive and interceptive orthodontics during the early deciduous tooth or teeth loss in the developing dentition [3, 4]. Different types of space maintainer have been designed in the last three decades. Although these space maintainers are classified as totally removable or fixed, all types are served for preventing the loss of arch perimeter [3, 5–8]. One can clearly see that while main advantage of fixed type space maintainer is directly related to no need for patient compliance [5], there are a few disadvantages as need for a laboratory process, long chair time, and patient discomfort due to taking impressions [6, 9]. Therefore, according to the individual need, lack of measurement space length, and limited adjustability [10–12], clinicians should provide a stone model of conventional space maintainers.

The aim of this study is to present a new adjustable Cise space maintainer for preventive orthodontic applications.

## 2. Materials and Methods

**Figure 1:**
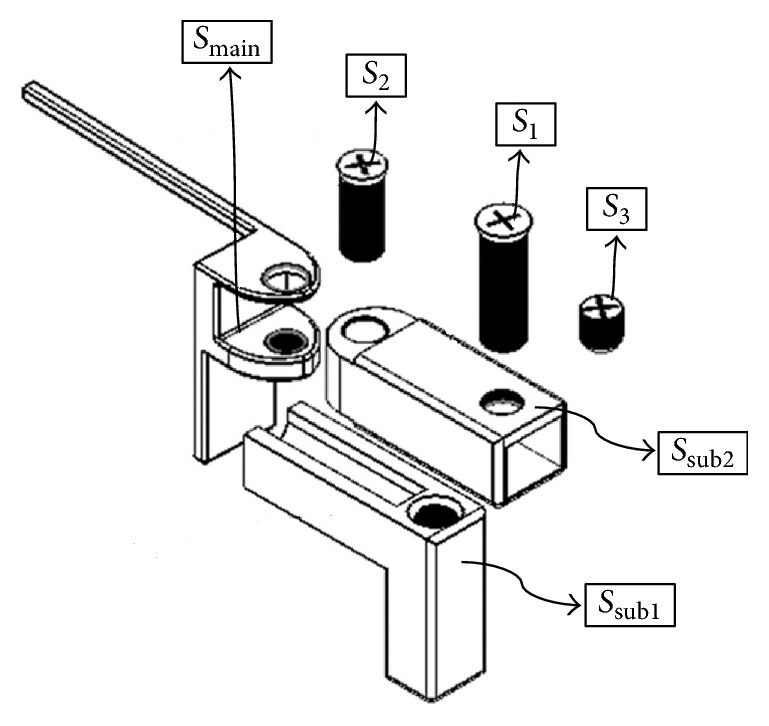
Main parts of the new adjustable Cise maintainer.

As seen in Figure 1, the new adjustable Cise maintainer consists of a main structure (*S*_main_) and two substructures (*S*_sub1_ and *S*_sub2_) with three screws (*s*_1_, *s*_2_, and *s*_3_). Material type of the maintainer is AISI 301 type stainless steel (DIN 1.4310) sheet with thickness of 0.6 mm. For the structural analysis, the material is assumed to be linear and isotropic (*ρ* = 7.81 × 10^−6^ kg/mm^3^, *E* = 200 GPa, and *ν* = 0.285).

**Figure 2:**
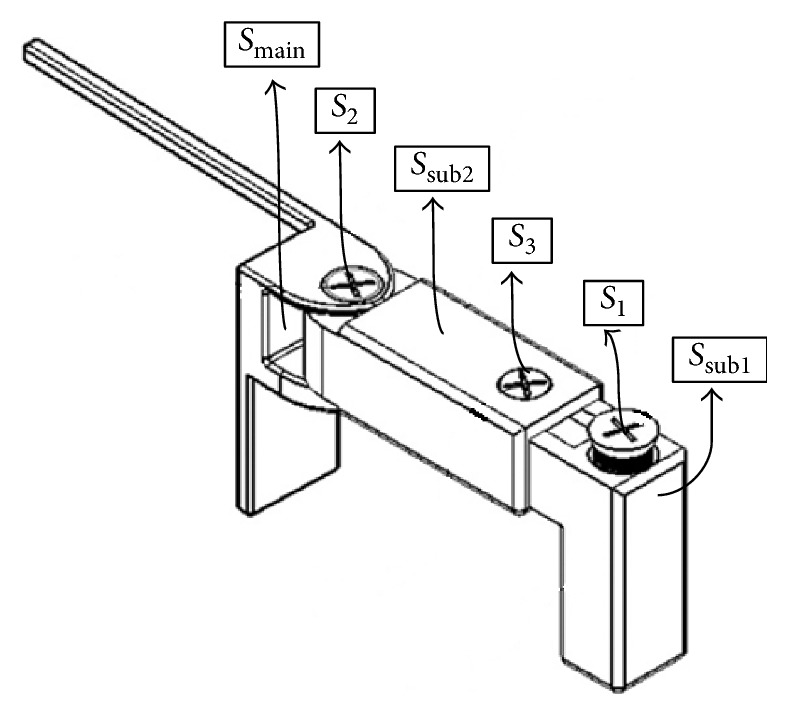
The new adjustable Cise space maintainer.

Three design steps of the maintainer are as follows:

- Substructure 2 is mounted to substructure 1 by using screw 3 (see Figure 2).
- The two-substructure part is completed by placing the main screw, namely, screw 1 (see Figure 2).
- The adjustable maintainer ends up with adding main structure to the two-substructure part with screw 2 (see Figure 2).

**Figure 3:**
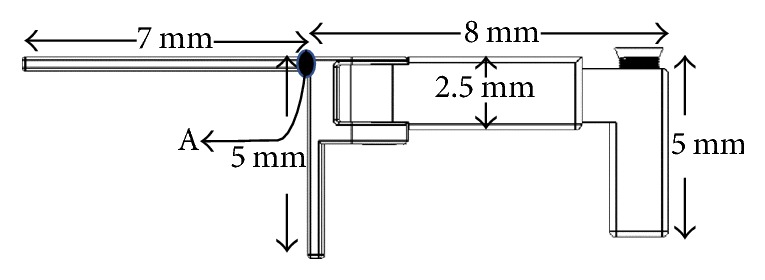
Geometrical representation of the adjustable Cise space maintainer.

Geometrical properties of the maintainer are given in Figure 3. In computational procedure, general-purpose finite element code MSC. Marc (v2014, MSC Software, Santa Ana, CA, USA) is used. It is assumed that the maintainer is under plane stress condition. Four-node linear plane stress element (full integration 3) is considered.

Moreover, in this structural analysis, strain energy density (SED) may be taken into account with an expression as

(1) $$\begin{array}{c} U_{i}=\overset{\varepsilon_{ij}}{\underset{0}{\int }}\sigma_{ij}\;d\varepsilon_{ij}. \end{array}$$

In (1), *σ*_*ij*_ and *ε*_*ij*_ are stress and strain components, respectively (*i*, *j* = 1–3). In particular, SED may be the main parameter for the thin structures.

## 3. Results

**Figure 4:**
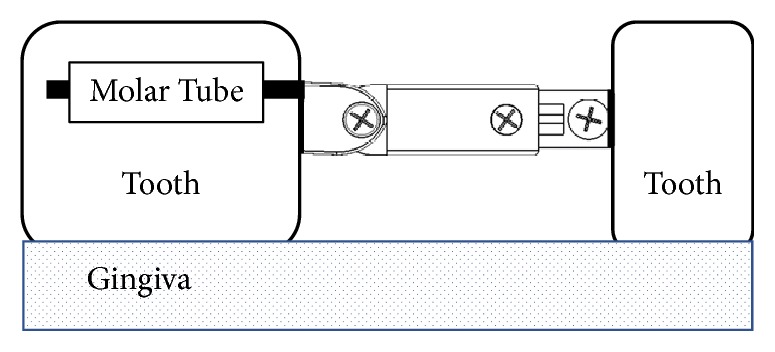
Application model for the adjustable Cise space maintainer.

**Figure 5:**
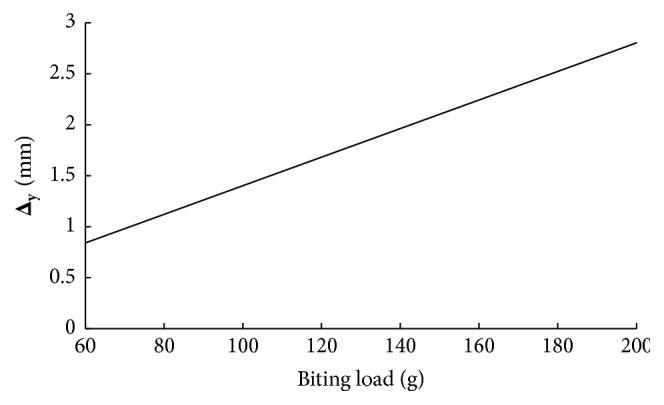
Variation of displacement in *y*-direction at A with biting force.

**Figure 6:**
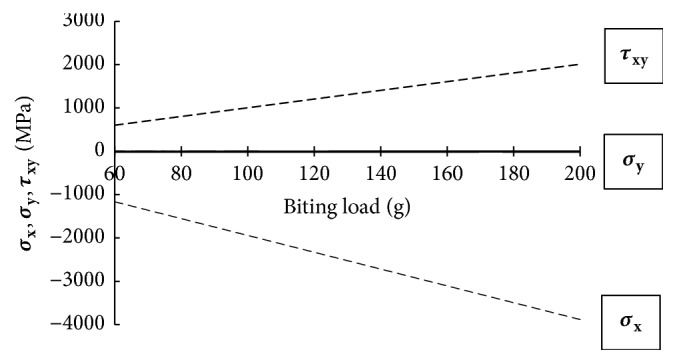
Variations of stresses **σ**_**x**_, **σ**_**y**_, and **τ**_**xy**_ at A with biting force.

**Figure 7:**
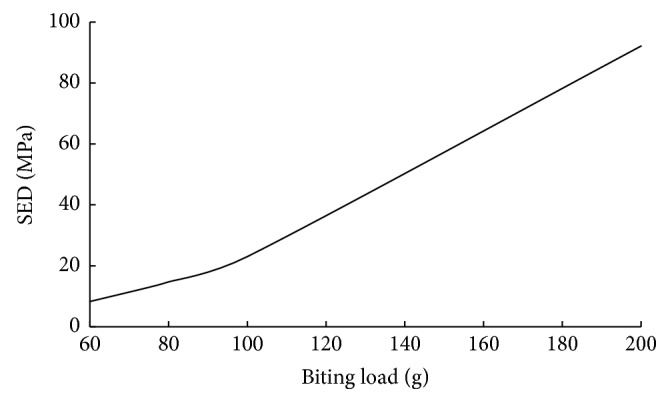
Variation of SED at A with biting force.

Regarding the critical point A and the application model for the Cise space maintainer given in Figures 3 and 4, respectively, linear variations of major displacement in *y*-direction (Δ_**y**_) and stresses (**σ**_**x**_, **σ**_**y**_, and **τ**_**xy**_) under biting load applied to the surface of the two-substructure part in *z*-direction are presented in Figures 5 and 6. Statically, on one hand, one can conclude that increasing biting force increases values of Δ_**y**_, **σ**_**x**_, and **τ**_**xy**_. In addition to that, variations of stress field indicate that **σ**_**y**_ is the minor stress element for the structural stability analysis. On the other hand, Figure 7 says that increasing biting load replaces characteristic linear behavior of SED with nonlinear behavior of SED up to the load of 100 g and then again shows linear behavior to the load of 200 g

## 4. Discussion

**Figure 8:**
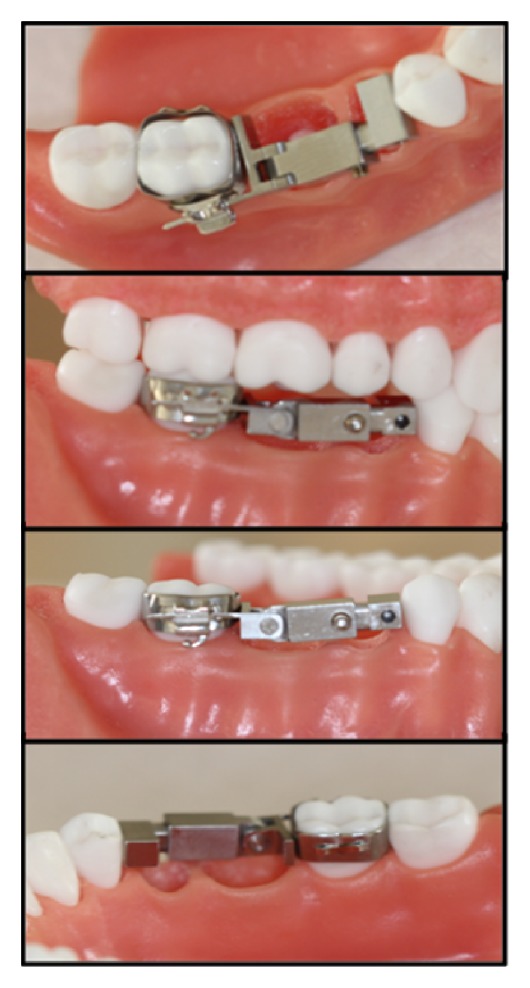
First prototype (2x magnification) of the adjustable Cise space maintainer on model.

Almost two-thirds of early loss of second primary molars and nearly half of the prematurely lost first primary molars can cause loss of space. This space lost can be the main factor of orthodontic crowding and/or impaction of permanent premolars. At some situation, anterior crowding or ectopic eruption of canine tooth due to the mesial movement of posterior teeth seems to be main problem [13, 14]. At this early loss, different types of space maintainers can be used [6, 9, 15]. Patient discomfort and need for patient compliance are main disadvantages of removable space maintainers. Some hygiene problems and periodontal problems can be seen during removable space maintainer therapy. At mixed dentition, patients are continuing to grow so the dimensions of arch length are changing. So, there is a need to make new removable space maintainer [6, 11, 12]. This means consuming more time and more money. The new design (see Figure 8) proposed, namely, three-dimensional adjustable fixed appliance, allows direct application to mouth. It can be placed by inserting its posterior part into the bondable orthodontic tube or band. Its length and location can be adjustable for individual patient arch shape and space dimensions with respect to uprighting the first molar and/or distal movement of molars. Moreover, some tooth cleaning and periodontal problems can be seen while using conventional fixed space problems [16–18]. Cise space maintainer can be removed from its location by a clinician. By the way, the periodontal problems can be treated.

Indirect-bonded space maintainer, which is one of the modern space maintainers, is a newly designed and presented appliance. Its properties are very good. However, this space maintainer can represent lower adjusting capacity than that of the Cise space maintainer. Some wire bends are to be done for adapting it to mouth [12]. The Cise space maintainer can be adjusted by screw 1, screw 2, and screw 3. Screw 2 connection can be used for vertical adaptation. Screw 3 connection can change the mesial-distal length of Cise space maintainer. Horizontal width adaptation can be done by adjusting the length of screw 1. Free three-dimensional adjustment without wire bending of the Cise space maintainer is one of the main advantages. While using the Cise space maintainer, there is no need for the bonding of mesial tooth (permanent or deciduous tooth); this situation is very advantageous on growing and changing dentition.

**Figure 9:**
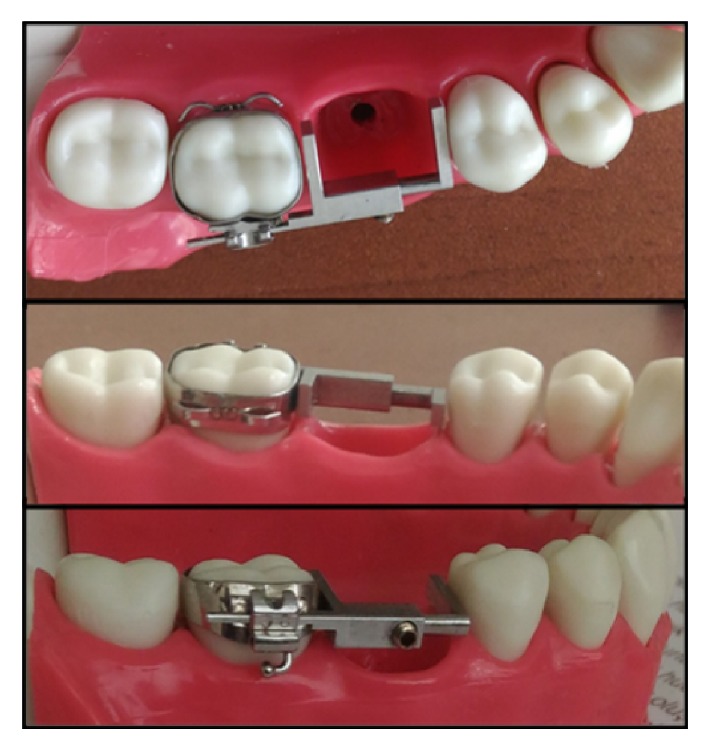
Simple form of the adjustable Cise space maintainer on model.

In recent studies, new approaches have been described and then related products have been considered as fixed space maintainers. Generally, fiber-reinforced composite materials are used for producing a space maintainer. Consequently, this type of space maintainer application is found to be more successful than conventional space maintainer at some aspects. This new approach possesses significantly higher patient acceptability and cost effectiveness. Additionally, on one hand, space maintainer with fiber-reinforced composite shows similar clinical performance to that of conventional band and loop space maintainer. On the other hand, its adjustable properties are lower than those of the Cise space maintainer. In other words, space maintainer made by fiber-reinforced composite is not easily adjustable and is in need of reconsidering after jaw growth [19]. The simple form of Cise space maintainer proposed can also allow adjusting different positions of newly erupted teeth comfortably. Another advantage of the maintainer is that it can be autoclaved and sterilized. In other words, the simple form of maintainer (see Figure 9) may be used for different patients with paying less health fee. This point is very important for the public dental health in especially underdeveloped countries.

Material type of the adjustable Cise space maintainer is stainless steel, namely, AISI 301 (DIN 1.4310). It is obviously seen that, because of the availability of this type alloy, other anchorage system parts such as orthodontic wires, lingual bars, and palatal bars are widely produced by AISI 301 type steel [20].

In dental biomechanics, computational difficulty emerges while determining an acceptable model to give accurate result. Additionally, linear elastic structural analysis is generally considered so that force-displacement results are much less sensitive to mesh quality. In such analysis, SED possesses two main functional roles, namely, dental rigidity [21] and tooth stability [22]. Consequently, in this study, Figure 7 indicates that (i) SED plays an important role to determine critical biting load capacity and (ii) the bilinear behavior says that adjustable Cise maintainer is one of the flexible, patient-friendly designs in orthodontic applications.

## 5. Conclusions

This new adjustable design proposed may be preferred in clinical trials. Structural analysis shows that the space maintainer is stable and is used for maintaining and/or regaining the space which arouses early loss of molar tooth. It may be concluded that future clinical studies are to be planned to use this type of design at routine preventive orthodontic practice.
